# Diversity of Neural Precursor Cell Types in the Prenatal Macaque Cerebral Cortex Exists Largely within the Astroglial Cell Lineage

**DOI:** 10.1371/journal.pone.0063848

**Published:** 2013-05-28

**Authors:** Christopher L. Cunningham, Verónica Martínez-Cerdeño, Stephen C. Noctor

**Affiliations:** 1 Neuroscience Graduate Program, University of California Davis, Davis, California, United States of America; 2 Institute for Pediatric Regenerative Medicine, Shriners Hospital for Children of Northern California, Sacramento, California, United States of America; 3 Department of Pathology and Laboratory Medicine, School of Medicine, University of California Davis, Sacramento, California, United States of America; 4 MIND Institute, School of Medicine, University of California Davis, Sacramento, California, United States of America; 5 Department of Psychiatry and Behavioral Sciences, School of Medicine, University of California Davis, Sacramento, California, United States of America; Seattle Children's Research Institute, United States of America

## Abstract

The germinal zones of the embryonic macaque neocortex comprise the ventricular zone (VZ) and the subventricular zone (SVZ). The mammalian SVZ is subdivided into an inner SVZ and an outer SVZ, with the outer SVZ being particularly large in primates. The existence of distinct precursor cell types in the neocortical proliferative zones was inferred over 100 years ago and recent evidence supports this concept. Precursor cells exhibiting diverse morphologies, patterns of transcription factor expression, and fate potential have been identified in the neocortical proliferative zones. Neurogenic precursor cells are thought to exhibit characteristics of glial cells, but the existence of neurogenic precursor cells that do not share glial specific properties has also been proposed. Therefore, one question that remains is whether neural precursor cells in the prenatal neocortex belong within the astroglial cell class, as they do in neurogenic regions of the adult neocortex, or instead include a diverse collection of precursor cells belonging to distinct cell classes. We examined the expression of astroglial markers by mitotic precursor cells in the telencephalon of prenatal macaque and human. We show that in the dorsal neocortex all mitotic cells at the surface of the ventricle, and all Pax6+ and Tbr2+ mitotic cells in the proliferative zones, express the astroglial marker GFAP. The majority of mitotic cells undergoing division away from the ventricle express GFAP, and many of the GFAP-negative mitoses express markers of cells derived from the ventral telencephalon or extracortical sites. In contrast, a markedly lower proportion of precursor cells express GFAP in the ganglionic eminence. In conclusion, we propose that the heterogeneity of neural precursor cells in the dorsal cerebral cortex develops within the GFAP+ astroglial cell class.

## Introduction

The cerebral cortex is populated by a diverse array of neuronal and glial cell types that are produced by precursor cells in the perinatal proliferative zones. Regional differences in fate potential are responsible for some of this diversity. For example, precursor cells in the proliferative zones of the ventral forebrain produce most cortical interneurons [1], while precursor cells in the proliferative zones of the dorsal forebrain produce multiple subtypes of excitatory projection neurons [2], some interneurons [3], [4], astrocytes [5], and oligodendrocytes [6]. Temporal differences in fate potential also contribute to the diversity of cortical cell types, as neural precursor cells generate different neuronal subtypes in a sequential ‘inside-out’ order [7]–[9]. In addition, sublineages of Cux2+ and Cux2-negative radial glial cells in the dorsal cerebral cortex that appear to produce distinct subtypes of excitatory projection neurons have been identified [10]. The existence of distinct precursor cell types in the neocortical proliferative zones was proposed over 100 years ago. For example, Wilhelm His proposed that the spongioblasts (now called radial glia) and germinal cells (cells dividing at the surface of the lateral ventricle) in the neocortical proliferative zones had distinct origins and different fate potentials – with germinal cells responsible for generating cortical neurons [11]. Sauer later demonstrated that spongioblasts and germinal cells were actually the same cell type in different phases of the cell cycle [12]. Nonetheless, the concept that different cortical cell types derive from distinct precursor cell types remains appealing since it provides a parsimonious explanation for the diversity of cortical cell types. Rakic and colleagues provided support for this concept in the 1980 s when they reported that not all mitotic cells in the proliferative zones expressed GFAP, a marker of radial glial cells in the prenatal macaque [13]–[15]. Levitt et al. proposed that the GFAP-negative precursor cells could represent neural precursor cells while the GFAP-positive precursor cells would give rise to radial glia and later astrocytes [13], [14]. Work over the subsequent three decades has steadily filled in more details concerning the identity, function, and expression characteristics of precursor cells in the developing cerebral cortex. For example, radial glial cells, the primary precursor cell in the mammalian ventricular zone, were shown to be mitotic [16], and to produce cortical neurons [17]–[23]. These findings were consistent with work showing that astroglial cells produce neurons in neurogenic regions of the adult mammalian brain [24]–[27], and that all mitotic cells undergoing division at the surface of the lateral ventricle in rat express the radial glial marker vimentin [28]. Together these findings invite reconsideration of the longstanding hypothesis that neurons and glial cells derive from distinct precursor cell pools.

Further work has identified additional neural precursor cell types in the cortical proliferative zones. Bipolar radial glia that express Pax6 [29], were shown to produce multipolar secondary precursor cells, here called intermediate progenitor (IP) cells [22], that express Tbr2 [30], seed the SVZ [7], [22], and produce cortical neurons [21]–[23], [31], [32]. In addition, it has been shown that the mammalian SVZ has two distinct proliferative zones: an inner SVZ (iSVZ) and an outer SVZ (oSVZ) [33], [34], with a large proportion of neurogenic divisions occurring in the oSVZ of the non-human primate neocortex [33]–[35]. Previous work showed that radial glial cells translocate from the VZ through the SVZ in the prenatal cerebral cortex of monkeys [36], ferrets [37], humans [38], and rodents [22]. More recent work has shown that the shift of neurogenic precursor cells from the VZ to the SVZ in primates and other gyrencephalic and lissencephalic mammals occurs in part through the translocation of radial glial cells [34], [39]–[41], that remain neurogenic [39]. Radial glial cells that translocate away from the VZ into the SVZ maintain Pax6 expression [34], [39], [41], and some also express the oligodendrocyte lineage transcription factor Olig2 [34]. Together, these data show that neural precursor cells exhibiting distinct morphologies and molecular expression characteristics are distributed throughout the VZ, iSVZ and oSVZ. Finally, during neurogenesis additional precursor cell types that derive from the ventral telencephalon and extracortical sites are present in the dorsal cortical proliferative zones, including oligodendrocyte precursor cells [6], [42], [43], and mitotic microglial cells [44]. In light of recent advances in our understanding of neural precursor cell identity, function, and characteristics, we re-examined the issue of precursor cell heterogeneity in the proliferative zones of the developing primate neocortex. We examined all mitoses in the neocortex - those that divided at the surface of the ventricle and those that divided away from the ventricle. We focused on three populations of precursor cells in the dorsal macaque telencephalon: mitotic cells that expressed Pax6, mitotic cells that expressed Tbr2, and mitotic cells that expressed neither Pax6 nor Tbr2. We also compared precursor cells in the dorsal telencephalon to those in the ganglionic eminence (GE). We used immunohistochemistry and confocal microscopy to identify and phenotype mitotic cells, and quantified marker expression by precursor cells during neurogenic and post-neurogenic stages of cortical development. We show here that in the human and non-human primate dorsal telencephalon all mitotic cells at the surface of the ventricle express GFAP. We show that all Pax6+ mitotic cells, including all Pax6+ cells undergoing division at the surface of the ventricle and away from the ventricle, express GFAP, and that all Tbr2+ mitoses express GFAP. We also show that the majority of abventricular mitoses are GFAP+ during neurogenic stages of cortical development in macaque. We also show that in the GE, a lower proportion of precursor cells express GFAP. We find that most of the GFAP-negative precursor cells undergoing division away from the ventricle are Mash1/ASCL1+ neural precursor cells, Olig2+ oligodendrocyte precursor cells, or Iba1+ mitotic microglia. These data demonstrate clear differences in developmental programs operating in the dorsal versus ventral telencephalon in prenatal macaque. The data support the hypothesis that neurogenic precursor cells in the dorsal cerebrum are in the astroglial lineage, which is defined as the neuroepithelial radial glial astrocyte lineage [24], [45], [46]. We conclude that the heterogeneity of cortical neural precursor cells develops within this cell class, rather than alongside this cell class.

## Results

### Temporal Patterns of GFAP Expression in the Dorsal Neocortex during Embryonic Development of Macaque Monkeys

Vimentin antibodies label radial glial cells in both rodent [47], and macaque [48]. We have shown that phosphorylated vimentin (4A4) labels all precursor cells undergoing division at the surface of the lateral ventricle in rat [28] and also in macaque [34]. These data suggest that precursor cells in the rat and macaque VZ belong to a single class of glial cells that share in common vimentin expression. Consistent with this idea, it has been proposed that VZ precursor cells are in the glial lineage [24], [45], [46], [49]. Indeed, GFAP labels astroglial cells in the prenatal, postnatal, and adult macaque and human neocortex [15], [37], [50], including neural precursor cells in neurogenic regions of the adult neocortex [25], [26]. However, Rakic and colleagues have shown that while GFAP labeled radial glial cells in the prenatal macaque neocortex [15], not all mitotic cells in the macaque proliferative zones expressed GFAP [13], [14]. We hypothesized that these seemingly contradictory findings might be explained by species differences, regional differences, or perhaps differential expression of GFAP by the neural precursor cell types that are now known to populate the VZ, iSVZ and oSVZ in primates, such as Pax6-expressing radial glial cells and Tbr2-expressing intermediate progenitor cells [34], [39], [41], [49], [51]. We therefore tested which precursor cell types expressed GFAP in the developing macaque (*Macaca mulatta*) cerebral cortex, looking specifically for differential GFAP expression by mitotic precursor cells dividing at the surface of the ventricle versus those dividing in abventricular locations, or by mitotic precursor cells that expressed markers of the neuronal lineages (Pax6, Tbr2, ASCL1), versus those that expressed markers expressed by glial (Olig2) or myeloid lineages (Iba1).

**Figure 1:**
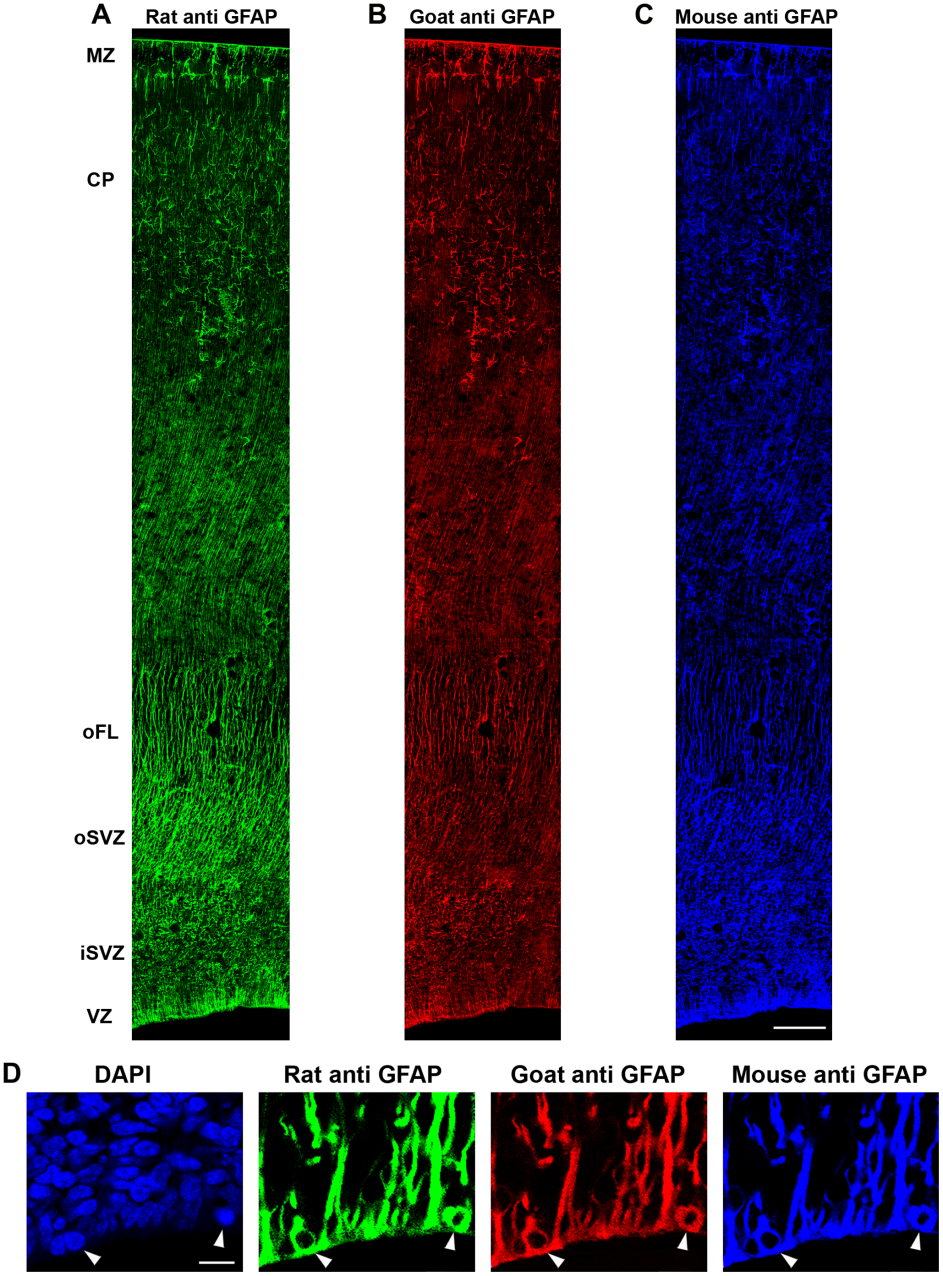
Multiple GFAP antibodies produce equivalent patterns of immunoreactivity in the developing cerebral cortex of macaque. *(* ***A–C*** *)* Panels show a coronal section of E80 macaque occipital cortex that was triple immunostained with three anti-GFAP antibodies. *(* ***A*** *)* Rat anti-GFAP from Virginia Lee, University of Pennsylvania. *(* ***B*** *)* Goat anti-GFAP from Santa Cruz. *(* ***C*** *)* Mouse anti-GFAP from Sigma-Aldrich. *(* ***D*** *)* Higher power magnification images of the ventricular surface from a coronal section of E80 macaque occipital lobe immunostained with the rat (green), goat (red) and mouse (blue) anti-GFAP antibodies. The left panel shows DAPI staining that labels the nuclei of all cells. Mitotic cells are indicated with white arrowheads. Each antibody labeled the same cells and cellular processes. Scale bar in ***C = ***200 µm, applies to all panels ***A–C***. Scale bar in ***D*** = 10 µm.

We first immunostained coronal sections of the neocortex obtained from macaque monkeys during neurogenic (embryonic day (E)50, E65, E80, E100) and post-neurogenic (E150) stages of cortical development [52], with anti-GFAP antibodies. We tested three anti-GFAP antibodies: a rat monoclonal antibody, a mouse monoclonal antibody, and a goat polyclonal antibody. Each antibody produced an equivalent pattern of immunoreactivity (IR) in the embryonic neocortex, and triple immunostaining confirmed that each antibody labeled the same cells and cellular processes (Figs. 1A & 1B). The rat anti-GFAP antibody produced quality labeling and allowed for convenient double, triple, and quadruple immunostaining, and was therefore selected for our studies.

**Figure 2:**
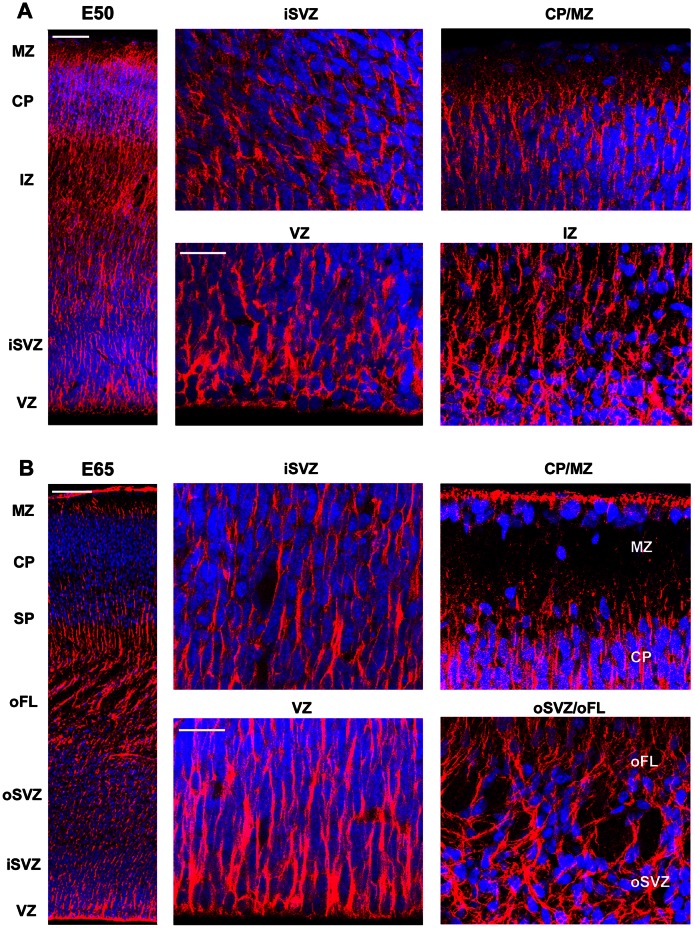
GFAP expression in the macaque neocortex during neurogenesis of lower layer cortical neurons. During neurogenesis of the layer 6 neurons *(* ***A*** *)* and layer 5 neurons *(* ***B*** *)* [52], GFAP immunostaining encircles the nuclei of ventricular surface cells and labels the pial fibers of radial glial cells. *(* ***A*** *)* E50 macaque occipital neocortex immunostained for GFAP (red) and costained with DAPI nuclear stain (blue). The left panel shows a coronal section of the E50 macaque occipital neocortex at low magnification. GFAP expression is observed across the cortical wall in the germinal zones, cortical plate and marginal zone. Panels to the right show higher magnification images of the ventricular zone (VZ), inner subventricular zone (iSVZ), intermediate zone (IZ) and cortical plate/marginal zone (CP/MZ). *(* ***B*** *)* E65 macaque occipital neocortex immunostained for GFAP (red) and costained with DAPI nuclear stain (blue). Left panel shows a coronal section of the E65 macaque occipital neocortex at low magnification. GFAP expression is observed across the entire cortical wall. Panels to the right show higher magnification images of the VZ, iSVZ, outer subventricular zone and outer fiber layer (oSVZ/oFL) and the CP/MZ. Scale bars in ***A*** and ***B***, upper left = 100 µm. Scale bars in ***A*** and ***B***, lower right = 25 µm.

**Figure 3:**
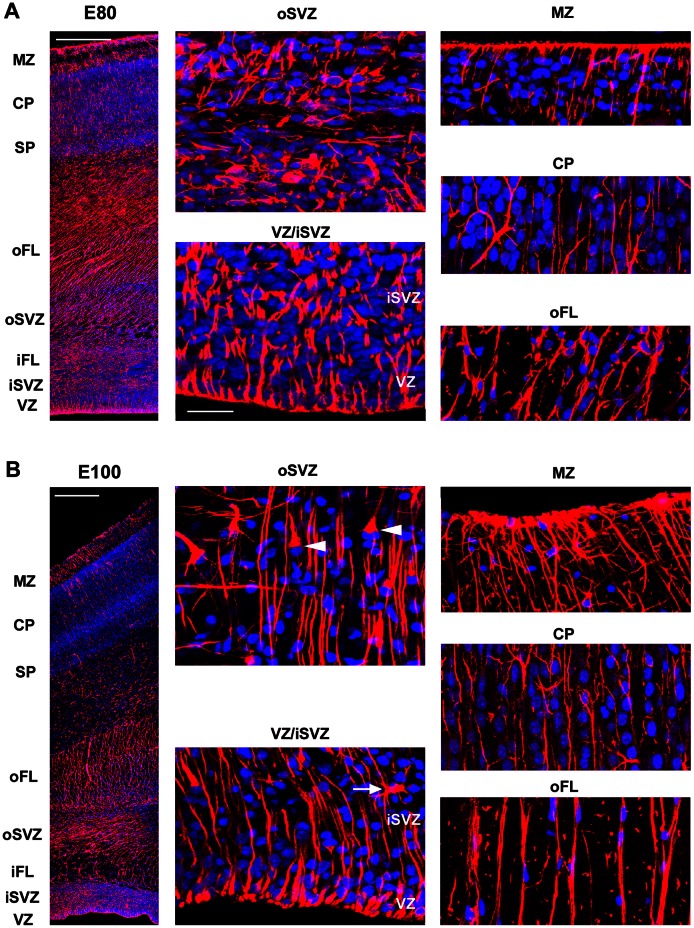
GFAP expression in the macaque neocortex during neurogenesis of upper layers. GFAP immunostaining is continuous along the surface of the ventricle, and dense GFAP-IR is present throughout the proliferative zones during neurogenesis of layer 4 neurons *(* ***A*** *)* and layer 2/3 neurons [52] *(* ***B*** *)*. *(* ***A*** *)* E80 macaque occipital neocortex immunostained for GFAP (red) and costained with DAPI (blue). Left panel shows a coronal section of the E80 macaque occipital neocortex at low magnification. Dense GFAP expression is observed throughout the germinal zones. Panels to the right show higher magnification images of the ventricular zone and inner subventricular zone (VZ/iSVZ), outer subventricular zone (oSVZ), outer fiber layer (oFL), cortical plate (CP), and marginal zone (MZ). *(* ***B*** *)* Coronal section of E100 macaque occipital neocortex immunostained for GFAP (red) and costained with DAPI nuclear stain (blue). At E100 GFAP expression is still dense in the cortical proliferative zones. Panels to the right show higher magnification images of the VZ/iSVZ, oSVZ, oFL, CP, and MZ. At E100 GFAP+ cells with the morphology of astrocytes were present in germinal zones (white arrow in VZ panel), and GFAP+ translocating radial glial cells were apparent (white arrowheads in oSVZ panel). Scale bars in ***A*** and ***B*** upper left = 500 µm. Scale bar in ***A***, VZ/iSVZ panel = 25 µm.

**Figure 4:**
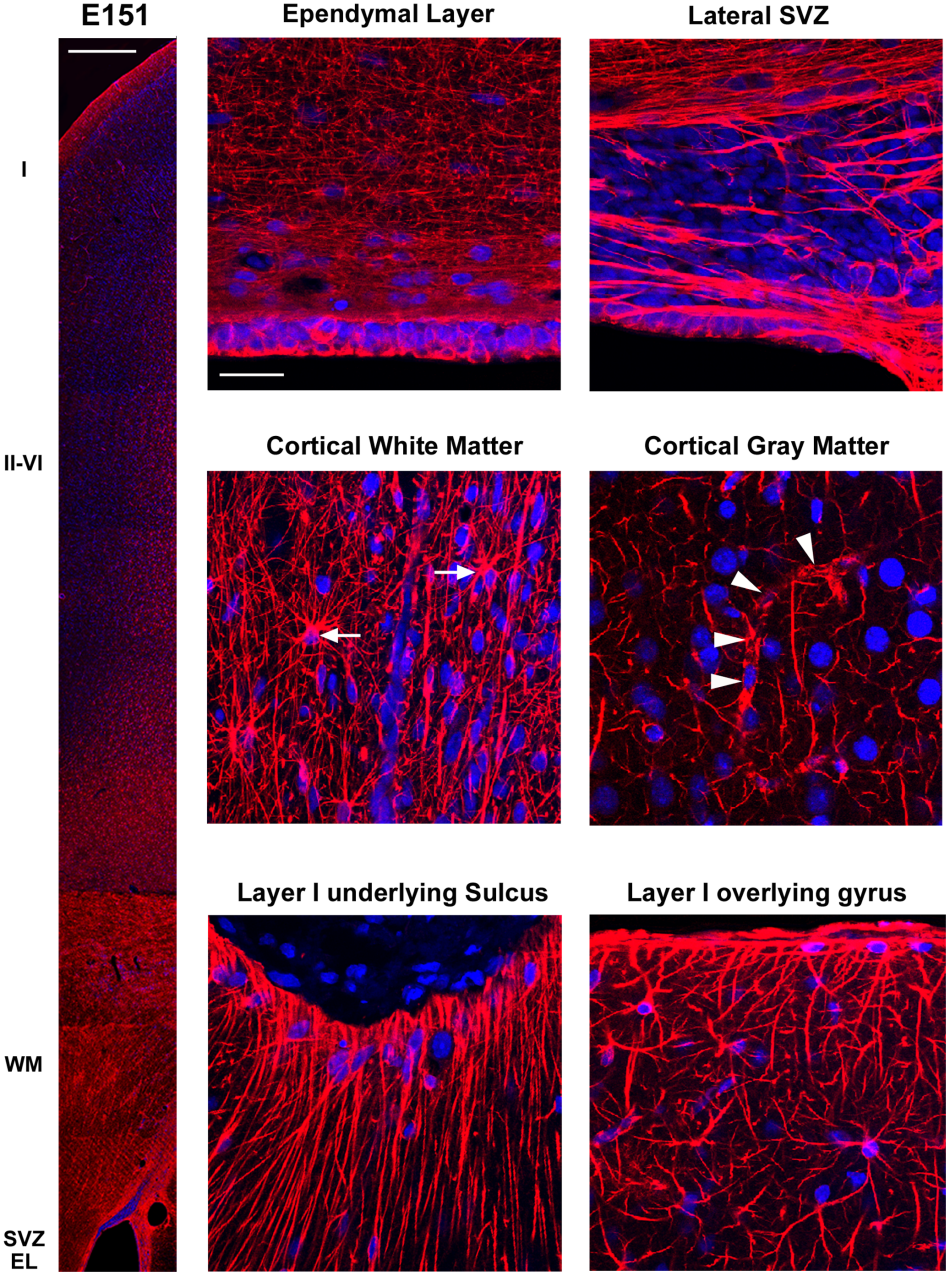
GFAP expression in the macaque neocortex after cortical neurogenesis. Panels show images taken from a coronal section of the E150 macaque neocortex in the occipital lobe immunostained for GFAP (red) and costained with DAPI (blue). Left panel shows a low magnification image. GFAP is expressed across the cortical wall in the ependymal layer (EL), subventricular zone (SVZ), white matter (WM), cortical gray matter, and pial surface. Right panels show higher magnification images of specific cortical zones. Many GFAP+ cells with the morphology of astrocytes were present in abventricular locations (white arrows). GFAP+ processes surrounded blood vessels (white arrowheads). Scale bar at left = 500 µm. Scale bar at right = 25 µm.

We next examined the temporal and regional expression patterns of GFAP in the prenatal macaque during neocortical development. At E50, GFAP-IR appeared to encircle the nuclei of all cells at the VZ surface and labeled the pial fibers of RG cells (Fig. 2A). At E65, E80, and E100 the overall pattern of GFAP-IR at the VZ surface was the same (Figs. 2B & 3), with decreasing numbers of mitoses at the surface at E80 and E100 as we have shown previously [34]. At E100, we noted more GFAP+ cells with the morphology of translocating radial glia and GFAP+ cells that resembled astrocytes (Fig. 3B). At E150, there was a band of GFAP+ ependymal cells at the edge of the ventricle, a dense band of GFAP+ cells and processes near the lateral ventricle, numerous GFAP+ astrocytes in the white matter and MZ, and a dense band of GFAP-IR at the pial surface. Blood vessels throughout the cortical wall appeared to be surrounded by GFAP+ elements (Fig. 4). In addition, we noted very different patterns of GFAP-IR in the marginal zone underlying sulci versus overlying gyri. Under sulci, GFAP+ fibers ran in dense bundles of fibers coursing perpendicular to the pial surface. In contrast, GFAP+ fibers in the marginal zone overlying gyri coursed in near random directions (Fig. 4). These findings show that the embryonic germinal zones of the dorsal telencephalon in prenatal macaque are characterized by strong GFAP expression, and that increasing numbers of GFAP+ translocating cells and GFAP+ transforming astroglial cells are observed across the neurogenic period.

### GFAP is Expressed by All Mitotic Cells at the Surface of the Ventricle in the Dorsal Neocortex of Prenatal Macaque throughout Neurogenesis

**Figure 5:**
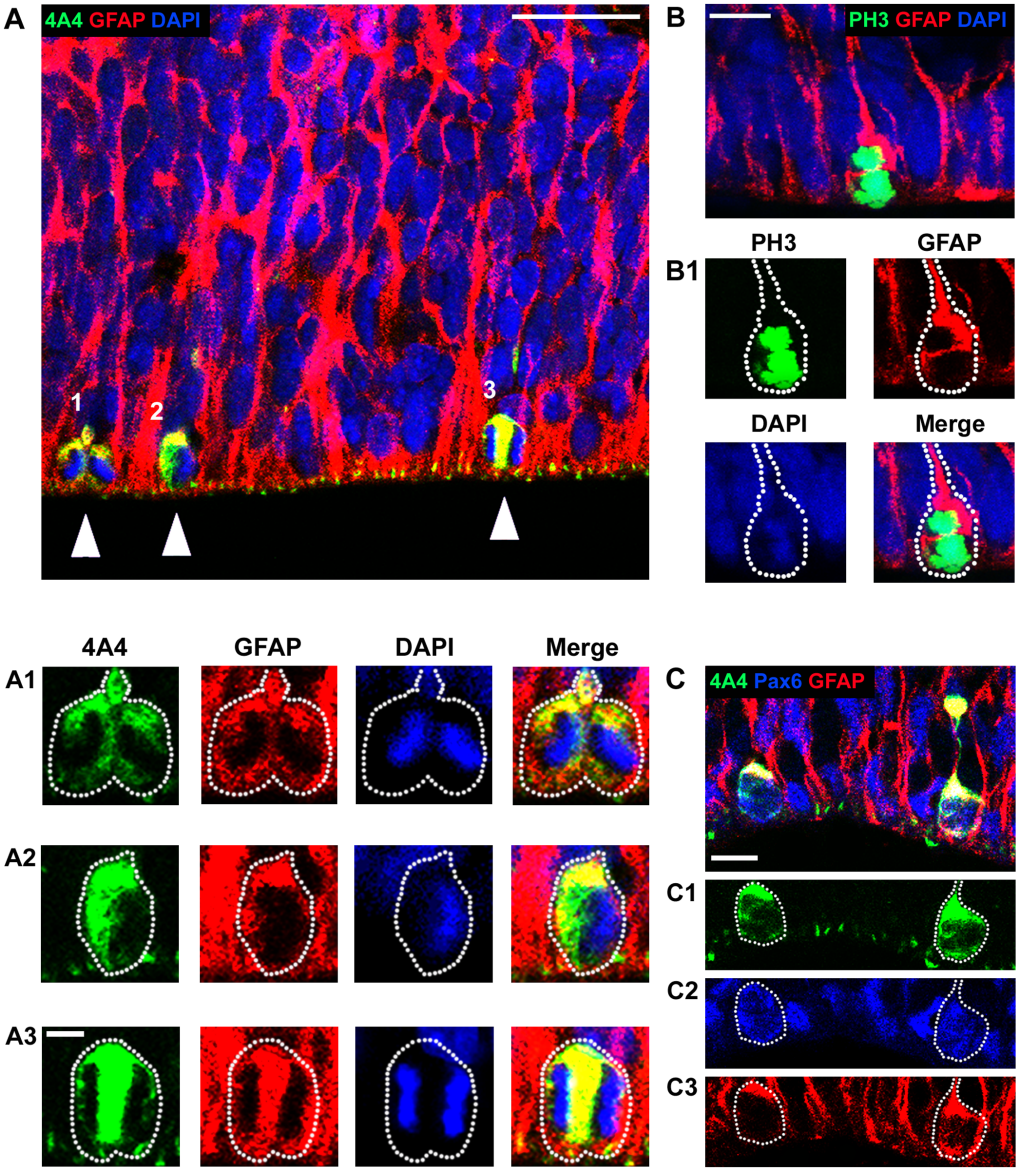
All mitotic cells at the ventricular surface of the macaque neocortical ventricular zone are GFAP+ and Pax6+ during cortical neurogenesis. Images show coronal sections of fetal macaque occipital neocortex immunostained with antibodies against phosphorylated vimentin (4A4, green) or phosphohistone H3 (PH3, green) to label all mitotic cells, and GFAP (red), and costained with DAPI (blue). *(* ***A*** *)* E65 macaque occipital cortex stained with 4A4 and GFAP antibodies. Panels ***A1***, ***A2***, and ***A3*** are magnified images of the numbered mitoses (white arrowheads) in Panel ***A*** showing colocalization of 4A4 and GFAP. *(* ***B*** *)* E80 macaque occipital cortex stained with PH3 and GFAP antibodies. Panel ***B1*** shows colocalization of PH3 and GFAP. *(* ***C*** *)* A coronal section of E80 macaque occipital cortex costained with antibodies against 4A4 (green), Pax6 (blue) and GFAP (red). All mitotic cells at the surface of the ventricle in the prenatal macaque neocortex expressed Pax6 and GFAP. Panels ***C1***, ***C2***, and ***C3*** show colocalization of 4A4, Pax6 and GFAP in surface mitoses. Scale bar in ***A*** = 25 µm, in ***A1*** = 5 µm, in ***B*** = 10 µm, in ***C*** = 10 µm.

**Figure 6:**
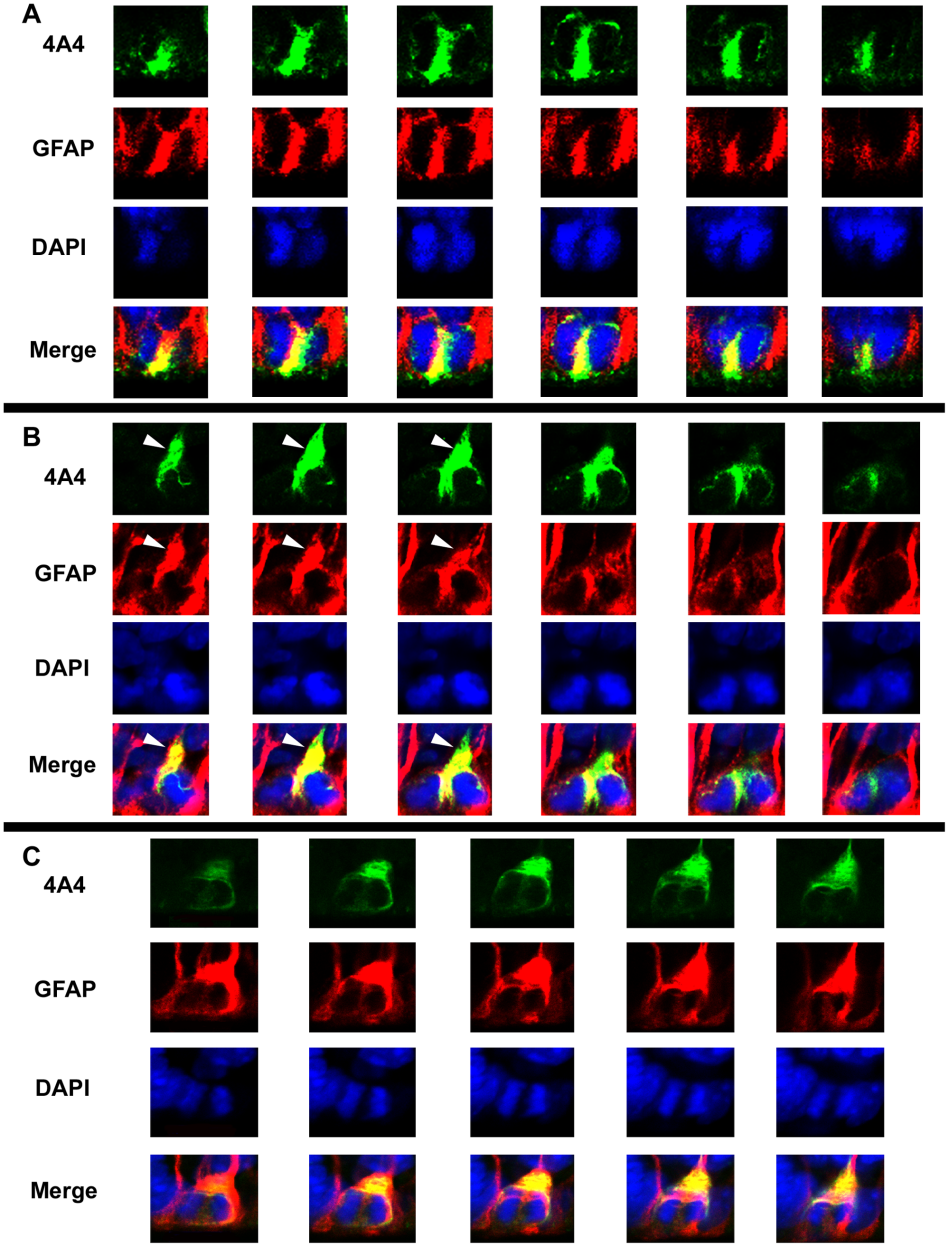
GFAP is expressed by all mitotic cells dividing at the surface of the ventricle in the macaque neocortical ventricular zone. Analysis of confocal Z-stack optical plane sections of ventricular surface mitoses at 0.5 µm steps from E65 *(* ***A*** *)* or E80 *(* ***B, C*** *)* macaque occipital cortex co-immunostained for 4A4 (green), GFAP (red) and DAPI (blue). Panels ***A–C*** show serial Z-stack images of the same cell, in each channel, from left to right. GFAP immunoreactivity was visualized in a thin rim of cytoplasm surrounding the nucleus, between sister chromatids, in the initial segment of the pial fiber (white arrowheads), and in the pial fiber. Yellow shows strong co-expression of the mitotic cell marker 4A4 and GFAP. Scale bar = 10 µm.

Our analysis of GFAP immunostaining at the ventricular surface showed dense immunoreactivity that appeared to label all cells at the surface of the lateral ventricle, where neurogenic radial glial cells divide. We therefore tested whether mitotic cells at the ventricular surface and in abventricular locations of the macaque occipital neocortex expressed GFAP, and if not, which cell-specific markers positively identified the GFAP-negative mitotic precursor cells. We double-immunostained neocortical macaque tissue from the occipital lobe at E50, E65, E80, E100, and E150 with anti-GFAP, and with antibodies that label mitotic cells (4A4 or phosphohistone H3). 4A4 labels all M-phase cells at the ventricular surface of rodent [28], [53] and macaque [34], and phosphohistone H3 (PH3) labels all mitotic cells in late G2 through early anaphase [34], [54]. We also counterstained the tissue with the DNA binding dye DAPI to confirm whether 4A4/PH3-IR cells were mitotic based on the pattern of chromatin staining [54], [55]. We imaged individual mitotic cells on a confocal microscope and quantified the proportion of VZ surface mitoses in the occipital lobe that expressed GFAP (Fig. 5). We found that GFAP was expressed by 100% of surface mitoses at E50 (n = 171 mitoses), E65 (n = 115), E80 (n = 101), and E100 (n = 26). We did not observe mitotic cells at the VZ surface at E150. We examined other cortical areas including frontal and somatosensory/motor cortex and obtained the same results. To ensure that mitotic cells were GFAP+ and not simply mitotic cells surrounded by GFAP+ fibers, we confocal imaged mitotic cells with a 60X or 100X objective at 0.5 µm Z-steps and examined each individual optical section for the presence or absence of GFAP expression in mitotic cells (Fig. 6). This approach showed that GFAP-IR in mitotic VZ cells was often located in a thin rim of cytoplasm that intimately enveloped the condensed chromatids, in the proximal segment of the pial fiber, and in the pial fiber.

GFAP isoforms that are expressed in subsets of astrocytes in the mature neocortex have been described, including GFAP-delta [56]. We therefore tested whether subsets of mitotic cells could be identified at the surface of the ventricle based on expression of GFAP isoforms. We immunostained tissue obtained from gestation week 19 (GW19) human neocortex with antibodies that label the GFAP-delta isoform and with pan-GFAP antibodies, but found that all mitoses at the ventricular surface of the GW19 human neocortex expressed both GFAP-delta and pan-GFAP. This finding is consistent with previous reports that all VZ cells in the human fetal neocortex expressed the GFAP-delta isoform and the c-terminus of GFAP-alpha, which is expressed by other GFAP isoforms [57].

**Figure 7:**
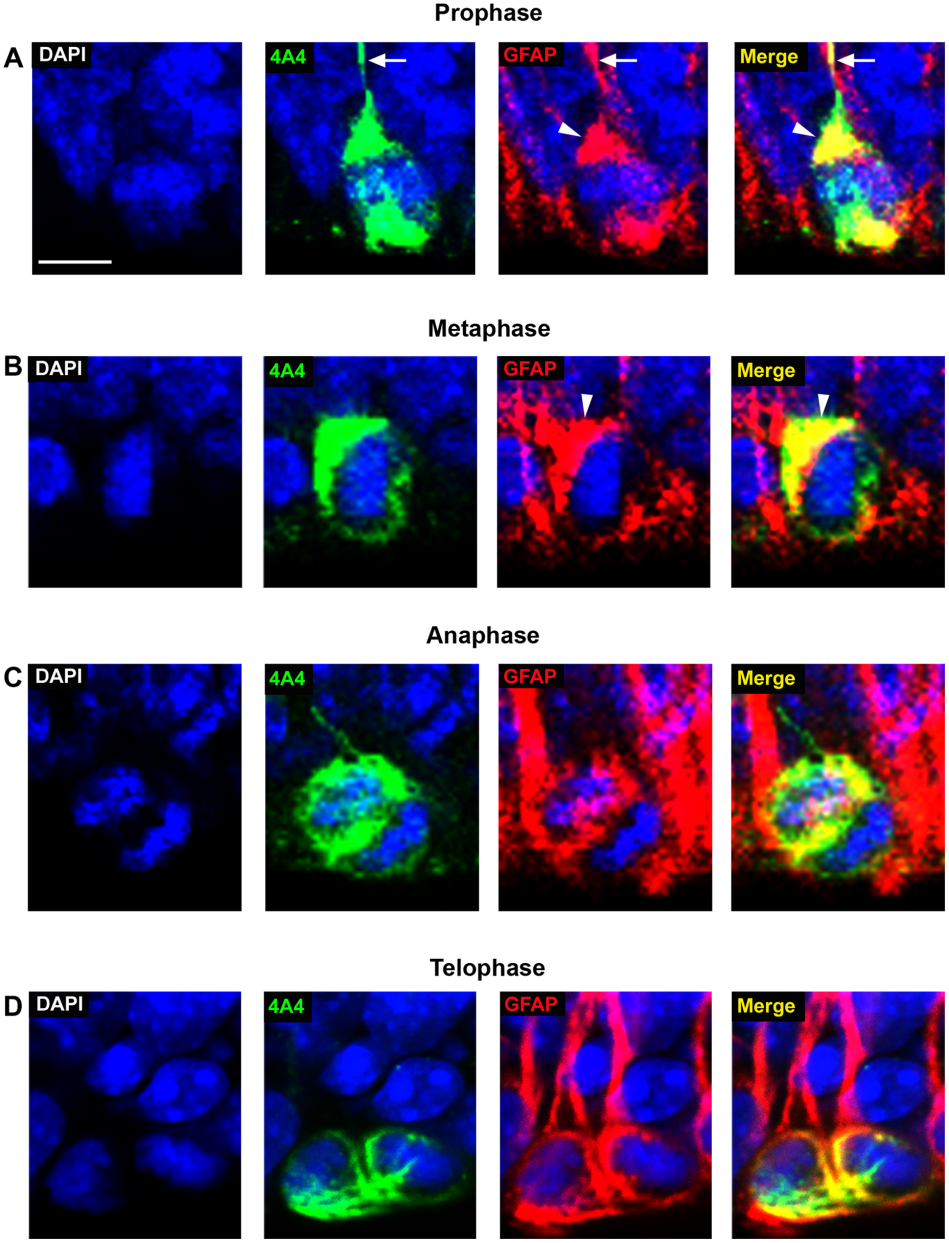
Distribution of GFAP in surface dividing VZ precursor cells during M-phase. Mitotic cells at the ventricular surface of the macaque occipital neocortex at E65 *(* ***A–C*** *)* and E80 *(* ***D*** *)* immunostained for the mitotic cell marker 4A4 (green) and GFAP (red), and costained with DAPI nuclear stain (blue). *(* ***A*** *)* 4A4+/GFAP+ prophase cell in the E65 macaque occipital cortex. During prophase there was strong GFAP immunoreactivity (IR) at the apical and basal poles of the cell. GFAP-IR often extended from the soma into the initial segment of the pial fiber on the basal pole of the cell to give the appearance of a “GFAP cap” (white arrowheads), and also labeled the pial fiber (white arrows). *(* ***B*** *)* 4A4+/GFAP+ metaphase cell in the E65 macaque occipital cortex. During metaphase GFAP encircled the nucleus in a relatively uniform pattern. The “GFAP cap” (white arrowheads) often appeared on one side of the condensed chromatin during metaphase. *(* ***C*** *)* 4A4+/GFAP+ anaphase cell in the E65 macaque occipital cortex. GFAP-IR surrounded the dividing chromosomes and was also present between the sister chromatids during anaphase. *(* ***D*** *)* 4A4+/GFAP+ telophase cell from E80 macaque occipital cortex. GFAP-IR surrounded the dividing sister chromatids of telophase cells and strong GFAP-IR was present in the cleavage furrow. Scale bar = 5 µm.

We next tested whether GFAP was expressed by surface dividing precursor cells throughout all stages of mitosis, including prophase, metaphase, anaphase and telophase (Fig. 7). During prophase there was a concentration of GFAP-IR at the apical and basal poles of the cell. GFAP-IR at the basal pole often extended from the soma into the pial fiber to give the appearance of a GFAP “cap” (white arrowheads, Fig. 7A). During metaphase, GFAP encircled the nucleus in a relatively uniform manner, but the GFAP “cap” often appeared on one side of the metaphase plate (white arrowhead, Fig. 7B). We interpreted this as a predictor for which sister chromatid would inherit the pial fiber after cell division was complete. During anaphase, GFAP-IR surrounded the dividing chromatids and was present between the sister chromatids (Fig. 7C). During telophase, GFAP-IR completely surrounded both sister chromatids and was strongly expressed at the base of the cleavage furrow (Fig. 7D). These data show that GFAP is expressed consistently throughout mitosis and that all mitotic cells at the surface of the ventricle expressed both 4A4 and GFAP (Fig. 7).

**Figure 8:**
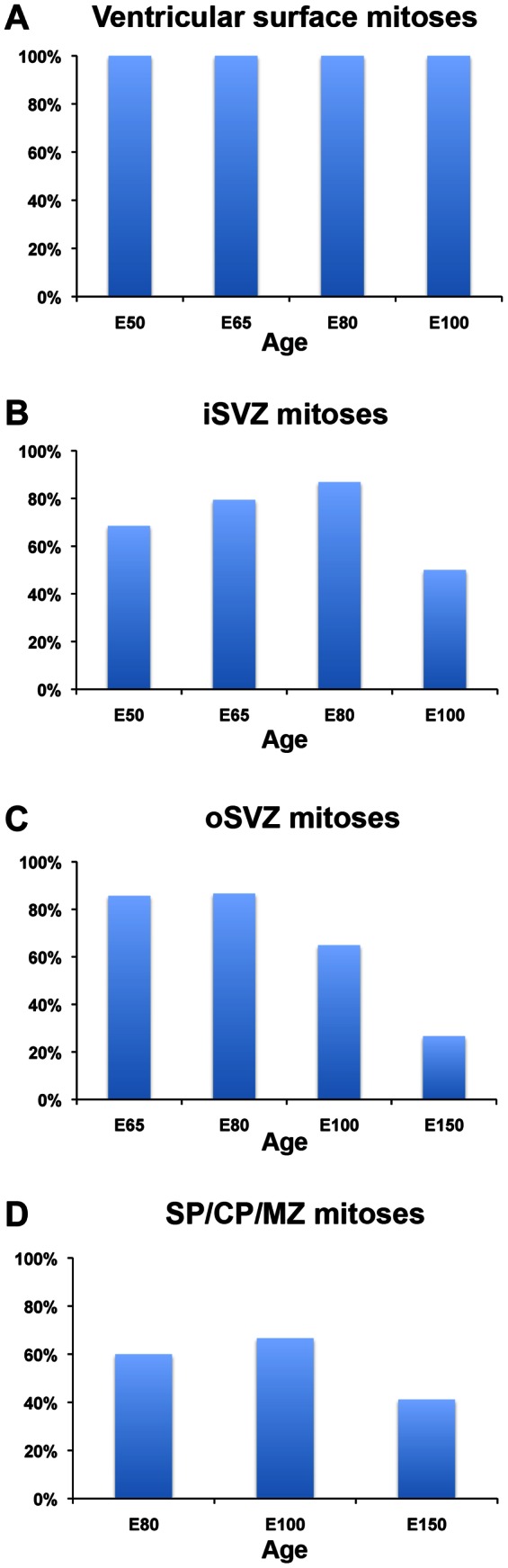
GFAP expression by 4A4+ mitotic cells in the macaque subventricular zone. Images show single optical plane sections from the inner subventricular zone (***A, B***), and outer subventricular zone (***C, D***) of the prenatal macaque. *(* ***A, B*** *)* 4A4+ (green)/GFAP+ (red) mitotic cells in the E65 inner subventricular zone nuclei stained with DAPI (blue). The vast majority of 4A4+ mitotic cells in the inner subventricular zone expressed GFAP. Insets at right show 4A4+/GFAP+ cells from numbered mitoses in the images at left. Merged panels show colocalization of 4A4 and GFAP (yellow) in the mitotic cells. *(* ***C*** *)* 4A4+ (green) GFAP+ (red) mitotic cells in the E65 outer subventricular zone. GFAP-immunoreactivity extended into the proximal portion of pial-directed processes of oSVZ mitoses (white arrow). Insets at right show 4A4+/GFAP+ cells from the image at left. *(* ***D*** *)* 4A4+ (green)/GFAP+ (red) mitotic cells in the E80 outer subventricular zone. Insets at right display 4A4+/GFAP+ cells from the image at left. The majority of 4A4+ mitotic cells in the outer subventricular zone expressed GFAP. GFAP-immunoreactivity often extended into the proximal portion of pial-directed processes of oSVZ mitoses (white arrow). Merged panels show colocalization of 4A4 and GFAP (yellow) in the mitotic cells. Scale bars in ***A–D*** = 20 µm, in ***A1–D2*** = 10 µm.

Precursor cells in the VZ express the transcription factor Pax6 [29], [30], but Tbr2-expressing precursor cells are also found in the VZ of rat [23] and macaque [34]. We therefore quantified the proportion of VZ surface mitoses that expressed Pax6 and GFAP in the occipital cortex and found that 100% of surface mitoses expressed both Pax6 and GFAP (n = 103 mitoses, Fig 5C). We did not observe any mitotic cells at the surface of the ventricle in macaque that expressed Tbr2 at any age (n = 194 mitoses). These data showed that in the macaque all mitotic cells at the dorsal surface of the lateral ventricle expressed GFAP throughout neurogenic stages of cortical development (Fig. 8A), and suggest that GFAP expression defines the population of astroglial precursor cells at the surface of the lateral ventricle in the embryonic neocortical macaque VZ.

### GFAP-positive and GFAP-negative Precursor Cells Undergo Division in the Abventricular Proliferative Zones of the Dorsal Neocortex in Prenatal Macaque

**Figure 9:**
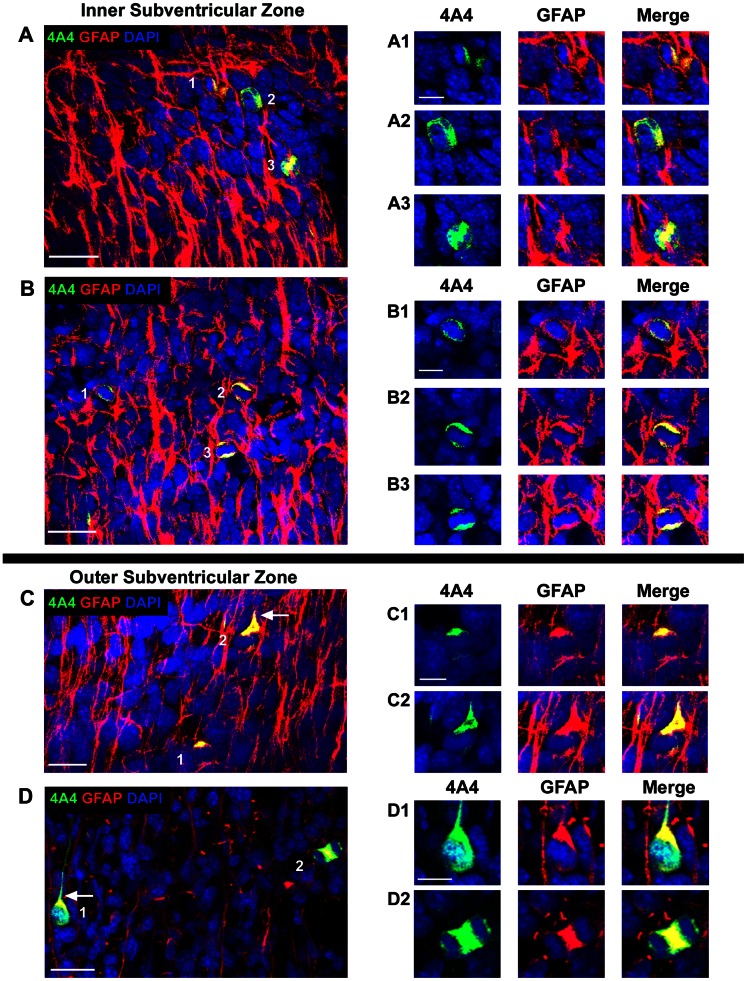
GFAP expression by 4A4+ mitotic cells in the marginal zone, cortical plate, and cortical gray matter during neurogenesis. Images in panels ***A–C*** show coronal sections of prenatal macaque monkey cerebral cortex immunostained for 4A4 (green) and GFAP (red), and stained with DAPI (blue). The majority of 4A4+ mitotic cells in extra-germinal zones expressed GFAP throughout neurogenesis. *(* ***A*** *)* 4A4+ mitotic cell co-expressing GFAP in the marginal zone (MZ) of E80 macaque occipital cortex. *(* ***B*** *)* 4A4+ mitotic cell expressing GFAP in the cortical plate (CP) of E100 macaque occipital cortex. Many 4A4+/GFAP+ mitotic cells in the CP had apparent astrocyte morphology. *(* ***C*** *)* 4A4+ mitotic cell expressing GFAP in the cortical gray matter of E100 macaque occipital cortex. Some 4A4+/GFAP+ cells had the morphology of translocating radial glia (top row). These cells were unipolar with a pial-oriented fiber that often had a large GFAP+ varicosity near the soma (white arrows). Scale bars in left panels *(* ***A–C*** *)* = 20 µm. Scale bars in 4A4 inset panels = 10 µm and apply to all inset panels.

**Figure 10:**
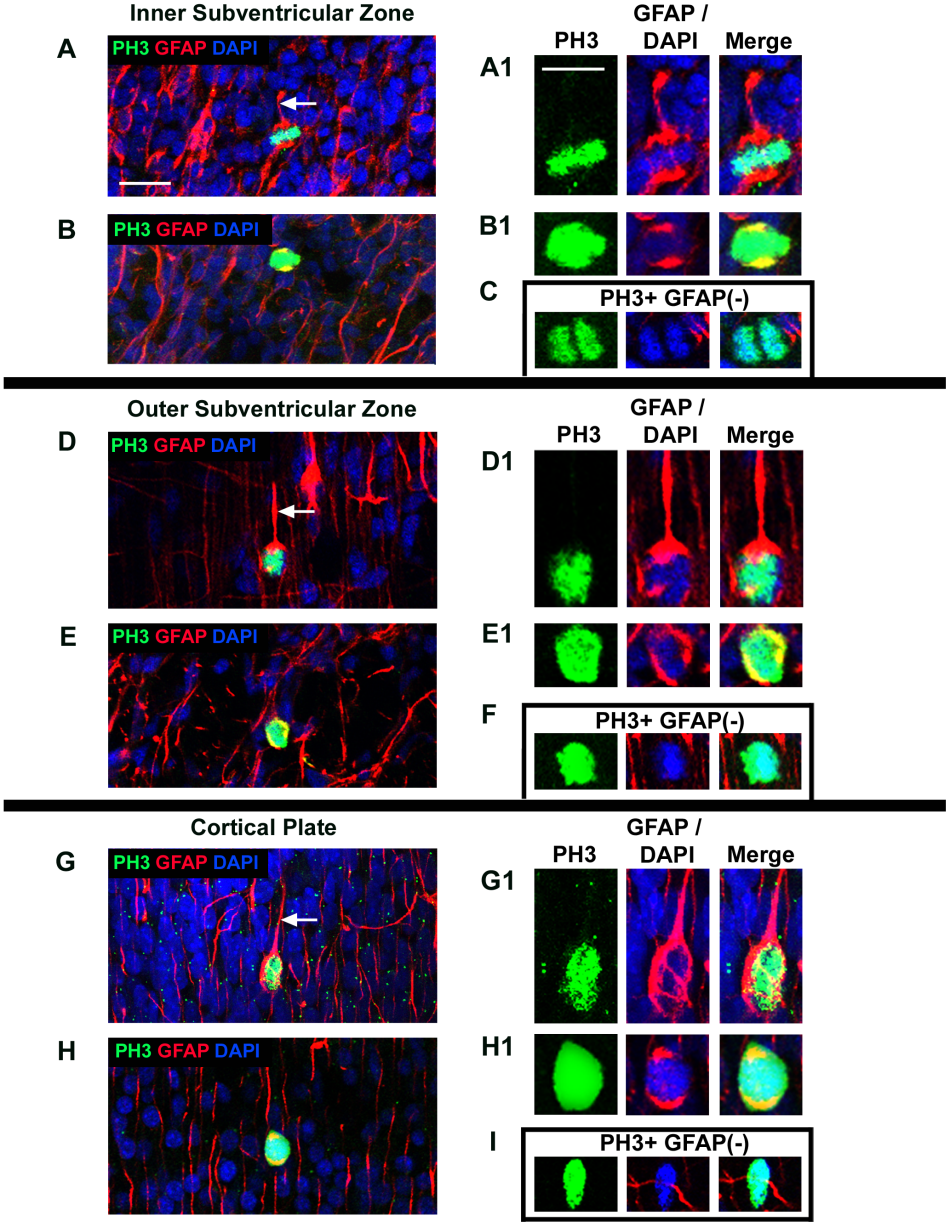
GFAP is expressed by the majority of PH3+ abventricular mitotic cells. *(* ***A–I*** *)* Coronal sections of the prenatal macaque cerebral cortex immunostained for PH3 (green) and GFAP (red), and stained with DAPI (blue). Most PH3+ mitotic cells in all layers of the developing cerebral cortex expressed GFAP *(* ***A–B, D–E, G–H*** *)*, but some PH3+ mitoses were GFAP-negative *(* ***C, F, I*** *)*. *(* ***A*** *)* PH3+/GFAP+ mitotic cell with a GFAP+ pial oriented process (white arrow) in the inner subventricular zone of E80 macaque occipital cortex. *(* ***A1*** *)* Images show colocalization (yellow) of PH3 and GFAP in the mitotic cell shown in panel ***A*** to the left. *(* ***B*** *)* Some PH3+/GFAP+ mitotic cells had no apparent GFAP+ processes but GFAP immunoreactivity encircled the nucleus. *(* ***B1*** *)* Images show colocalization (yellow) of PH3 and GFAP in the mitotic cell shown in panel ***B*** to the left. *(* ***C*** *)* Example of a PH3+ mitotic cell that did not express GFAP in the inner subventricular zone of E80 macaque occipital cortex. Note the lack of GFAP immunoreactivity between the sister chromatids. *(* ***D, E*** *)* PH3+/GFAP+ mitotic cells in the outer subventricular zone of E80 macaque occipital cortex. Many PH3+/GFAP+ mitotic cells had the morphology of translocating radial glia. The pial process was GFAP+ (white arrow). *(* ***D1, E1*** *)* Images show colocalization (yellow) of PH3 and GFAP in the mitotic cells shown in panels ***D*** and ***E*** to the left. *(* ***F*** *)* Example of a PH3+ mitotic cell that did not express GFAP in the outer subventricular zone of E80 macaque occipital cortex. *(* ***G, H*** *)* PH3+/GFAP+ mitotic cell in the cortical plate of E80 macaque occipital cortex. Many GFAP+ mitotic cells in extragerminal zones had the morphology of translocating radial glial cells with pial-oriented processes (white arrow). *(* ***G1, H1*** *)* Images show colocalization (yellow) of PH3 and GFAP in the mitotic cells shown in panels ***G*** and ***H*** to the left. *(* ***I*** *)* Example of a PH3+ mitotic cell in the gray matter of E150 macaque occipital cortex that did not express GFAP. Scale bar in ***A*** = 20 µm and applies to all images in left column. Scale bar in ***A1*** = 10 µm applies to all images in right column.

We next examined the expression characteristics of mitotic cells dividing away from the ventricle. We double immunostained tissue with markers for mitotic cells (4A4 or PH3) and GFAP to determine the proportion of abventricular mitoses that expressed GFAP in the iSVZ, the oSVZ, and more superficial structures during prenatal neurogenesis. The majority of mitoses in the iSVZ and oSVZ expressed GFAP during neurogenic stages (Fig. 9), but we observed both GFAP+ and GFAP-negative cells undergoing division in abventricular locations (Figs. 10C, 10F & 10I). Roughly 70 to 85% of mitotic cells in the iSVZ expressed GFAP between E50 and E80 (Fig. 8B). The proportion of GFAP+ mitotic cells in the iSVZ fell to 55% at E100, and the iSVZ was largely absent at E150. The oSVZ is not yet present in the macaque at E50 [34]. Between E65 and E80, approximately 60 to 85% of mitotic cells in the oSVZ expressed GFAP. The proportion of GFAP+ mitotic cells in the oSVZ fell to ∼55% at E100, and to 25% at E150 (Fig. 8C).

**Figure 11:**
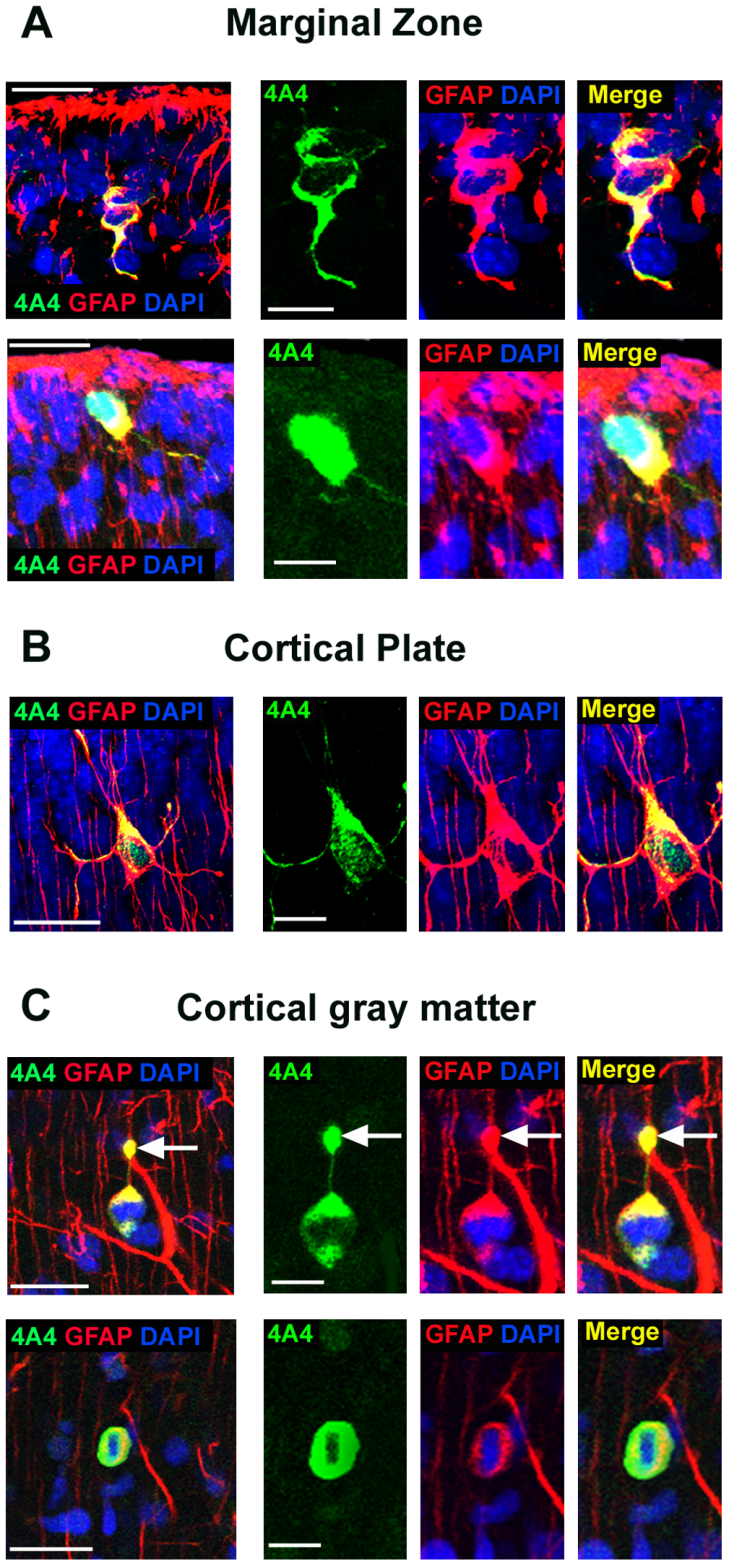
The proportion of mitotic cells that express GFAP in the prenatal macaque occipital cortex during neurogenic stages (E50, E65, E80, E100) and one postneurogenic stage (E150). Mitoses were identified in each proliferative zone and cortical layer by 4A4 immunoreactivity, PH3 immunoreactivity and/or condensed chromatin with DAPI. *(* ***A*** *)* 100% of all mitotic cells at the ventricular surface of the dorsal neocortex expressed GFAP during neurogenic stages. No surface dividing cells were detected at E150. *(* ***B*** *)* The majority of mitotic cells expressed GFAP in the inner subventricular zone (iSVZ) during cortical neurogenesis. The iSVZ was largely absent at E150. *(* ***C*** *)* The majority of mitotic cells in the outer SVZ (oSVZ) expressed GFAP during neurogenic stages of development. The oSVZ was not present at E50, and the proportion of mitotic cells that expressed GFAP at E150 fell to 25%. *(* ***D*** *)*. The majority of mitotic cells in extra-germinal zones expressed GFAP during neurogenesis, but not during post-neurogenic stages. SP, subplate; CP, cortical plate; MZ, marginal zone.

Mitotic cells are also present in structures superficial to the VZ and SVZ proliferative zones during neurogenic stages of primate cortical development, as shown in detail by Molnar and colleagues [58]. We examined the proportion of mitotic cells in the subplate, cortical plate, and marginal zone that expressed GFAP. There were very few extra-germinal mitoses between E50 and E65, but their numbers increased between E80 and E150. We detected mitoses in each cortical lamina (Fig. 11), with 40 to 75% of extra-ventricular mitoses expressing GFAP (Fig. 8D).

Our results confirm the previous finding of Levitt and Rakic by showing that not all neocortical precursor cells express GFAP [13], [14]. However, we found that all of the GFAP-negative precursor cells were located away from the ventricle.

### All Translocating Radial Glia and All Intermediate Progenitor Cells Express GFAP in the Dorsal Neocortex of Prenatal Macaques

We next determined the phenotype of the GFAP+ and GFAP-negative precursor cells dividing away from the surface of the ventricle. Recent work has demonstrated the presence of translocating radial glial (tRG) cells in the embryonic neocortex of rodents [22], [23], [34], [59], [60], carnivores [34], [40], [41], non-human lissencephalic primates [51], [61], non-human gyrencephalic primates [34], and humans [39], [41]. We hypothesized that since GFAP is not expressed by neurons [62], [63], the level of GFAP expression would be highest in precursor cells and lower in cells differentiating along the neuronal pathway, such as Tbr2+ IP cells.

**Figure 12:**
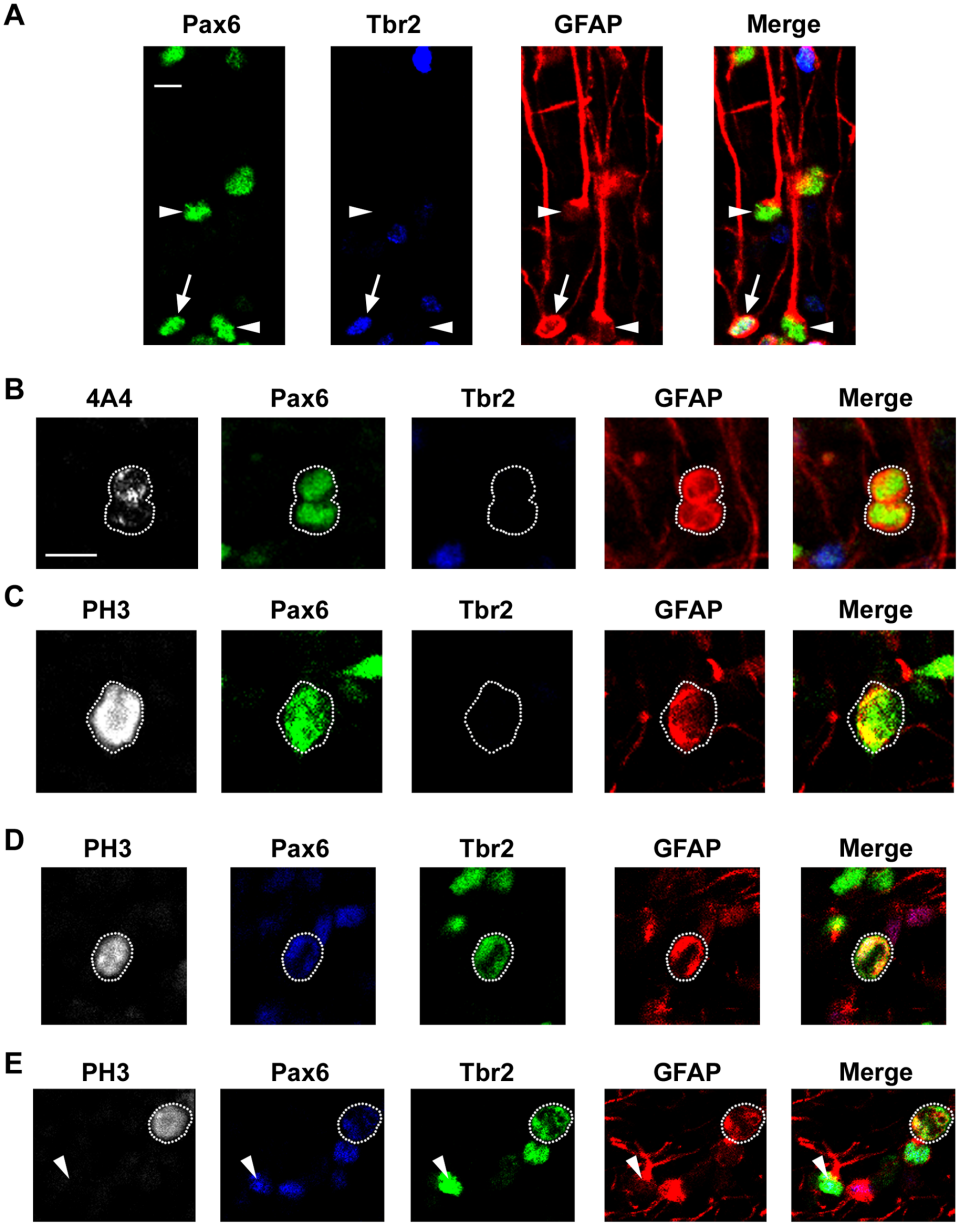
GFAP is expressed by all Pax6+ translocating radial glia (tRG) and all Tbr2+ intermediate progenitor cells. *(* ***A*** *)* A coronal section of the E80 macaque occipital cortex immunostained for Pax6 (green), Tbr2 (blue) and GFAP (red). Both tRG cells with GFAP+ pial fibers (Pax6+ Tbr2–, white arrowheads) and Tbr2+ intermediate progenitor cells (Tbr2+/Pax6+, white arrows) expressed GFAP. *(* ***B–E*** *)* Mitotic cells identified by 4A4 or PH3 (white) are outlined by white dotted lines. All images show single optical planes. *(* ***B, C*** *)* 4A4+ or PH3+ mitotic cells (white) that express Pax6 (green) but not Tbr2 (blue) expressed high levels of GFAP (red). The merge panel demonstrates co-expression of GFAP in Pax6+ mitotic cells (yellow). *(* ***D, E*** *)* PH3+ mitotic cells (white) that express Tbr2+ (green) and also Pax6+ (blue) co-expressed GFAP (red). The merge panel demonstrates co-expression of GFAP in Tbr2+ mitotic cells (yellow). We have previously shown that 90% of Tbr2+ cells in the macaque SVZ co-express Pax6 [34]. *(* ***D*** *)* An example of a mitotic Tbr2+ cell, indicated by white dotted lines, that is GFAP+. *(* ***E*** *)* A PH3+ mitotic cell (white), indicated by the white dotted line, expressing both Tbr2 (green) and GFAP (red). Some Tbr2+ cells expressed low levels of Pax6. An interphase Tbr2+ intermediate progenitor cell (PH3-negative, white arrowheads) also exhibits strong GFAP expression in the soma and cellular processes. Scale bars in ***A*** and ***B*** = 10 µm.

To determine cell-specific patterns of GFAP immunoreactivity in mitotic tRG and IP cells, we quadruple immunostained coronal sections of macaque tissue for 4A4 or PH3, GFAP, Pax6, and Tbr2. We identified mitotic tRG cells as 4A4/PH3+ cells that were Pax6+, Tbr2-negative, and possessed a visible 4A4+ pial fiber. We identified mitotic IP cells as 4A4/PH3+ that were Tbr2+/Pax6+, or Tbr2+/Pax6-negative as previously described [34]. We found that 100% of mitotic tRG cells in macaque expressed GFAP at high levels (Fig. 12, n = 35 cells) as previously reported for developing human neocortex [39], [41]. We also found that all Tbr2+ IP cells expressed GFAP. We examined the level of GFAP expression in the mitotic IP cells and found that approximately half expressed GFAP at strong levels (40/85 mitoses), and the remaining half expressed GFAP at weak levels (45/85 mitoses). These results show that all mitotic tRG cells and IP cells express GFAP. IP cells in the macaque may undergo symmetric proliferative divisions that expand their numbers to generate additional cortical neurons, as described in the human neocortex [39]. The strong and weak levels of GFAP expression by IP cells may reflect decreased GFAP expression by precursor cells as they divide and further differentiate along the neuronal pathway. Since all Tbr2+ and all Pax6+ precursor cells in the SVZ expressed GFAP, and since our results showed that between 15 to 45% of precursor cells in the SVZ did not express GFAP, our results indicate the presence of additional precursor cell types in the embryonic SVZ during neurogenic stages of macaque cortical development.

### GFAP-negative Precursor Cells in the Dorsal Neocortex Include ASCL1+ Neural Precursor Cells, Olig2+ Precursor Cells and Microglia

**Figure 13:**
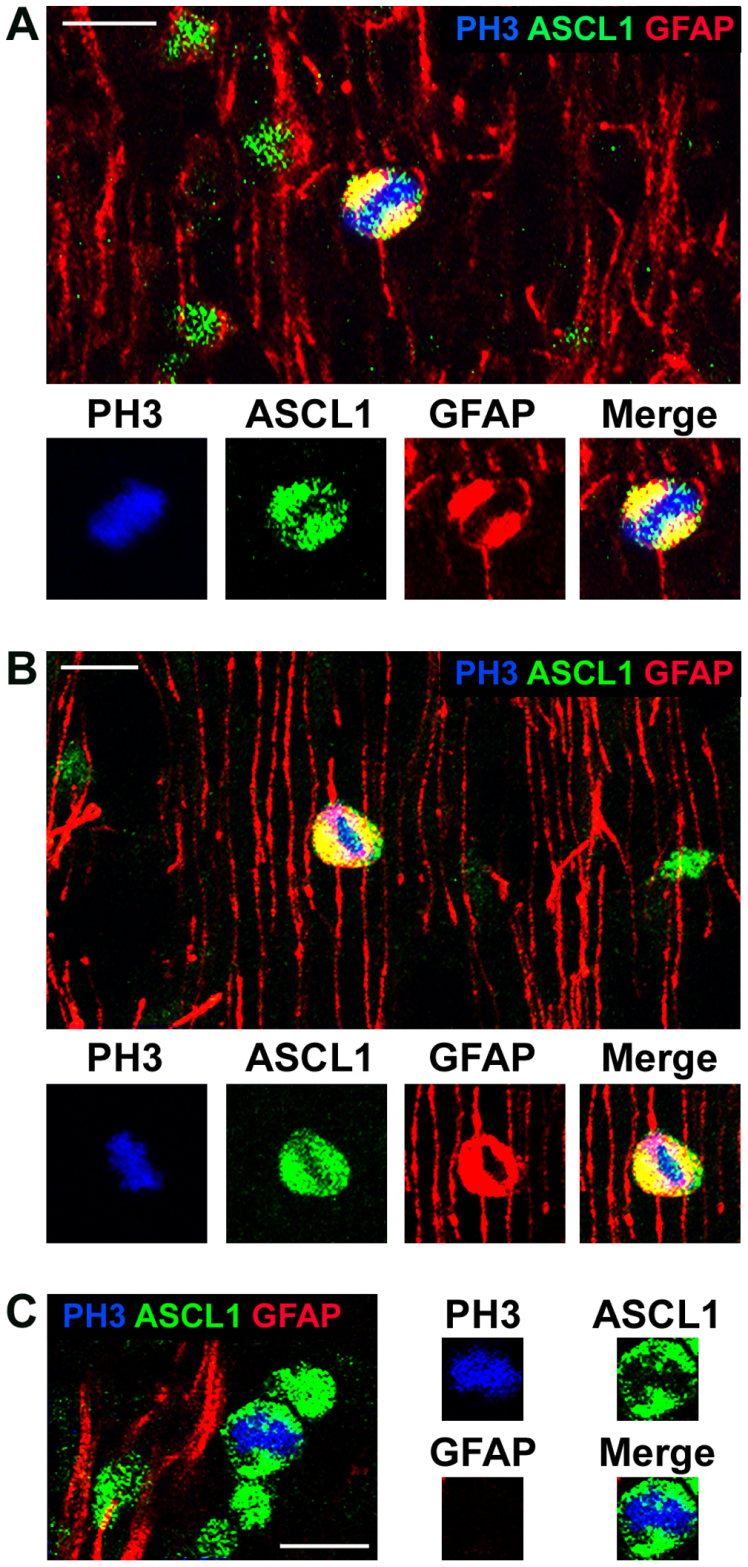
The majority of GFAP-negative mitoses in the prenatal macaque neocortex are Mash1/ASCL1+. Nevertheless, approximately 94% of ASCL1+ mitotic cells in the prenatal macaque cerebral cortex express GFAP. *(* ***A, B*** *)* Examples of ASCL1+/GFAP+ mitotic cells in the E80 macaque occipital cortex. Sections were immunostained for PH3 (blue), ASCL1 (green) and GFAP (red). *(* ***A, B*** *)* Examples of PH3+/ASCL1+/GFAP+ mitotic cells located in the outer subventricular zone. Inset panels below show immunoreactivity for each individual antibody. *(* ***C*** *)* An example of a PH3+/ASCL1+ mitotic cell that does not express GFAP in the subventricular zone. Scale bars = 10 µm.

**Figure 14:**
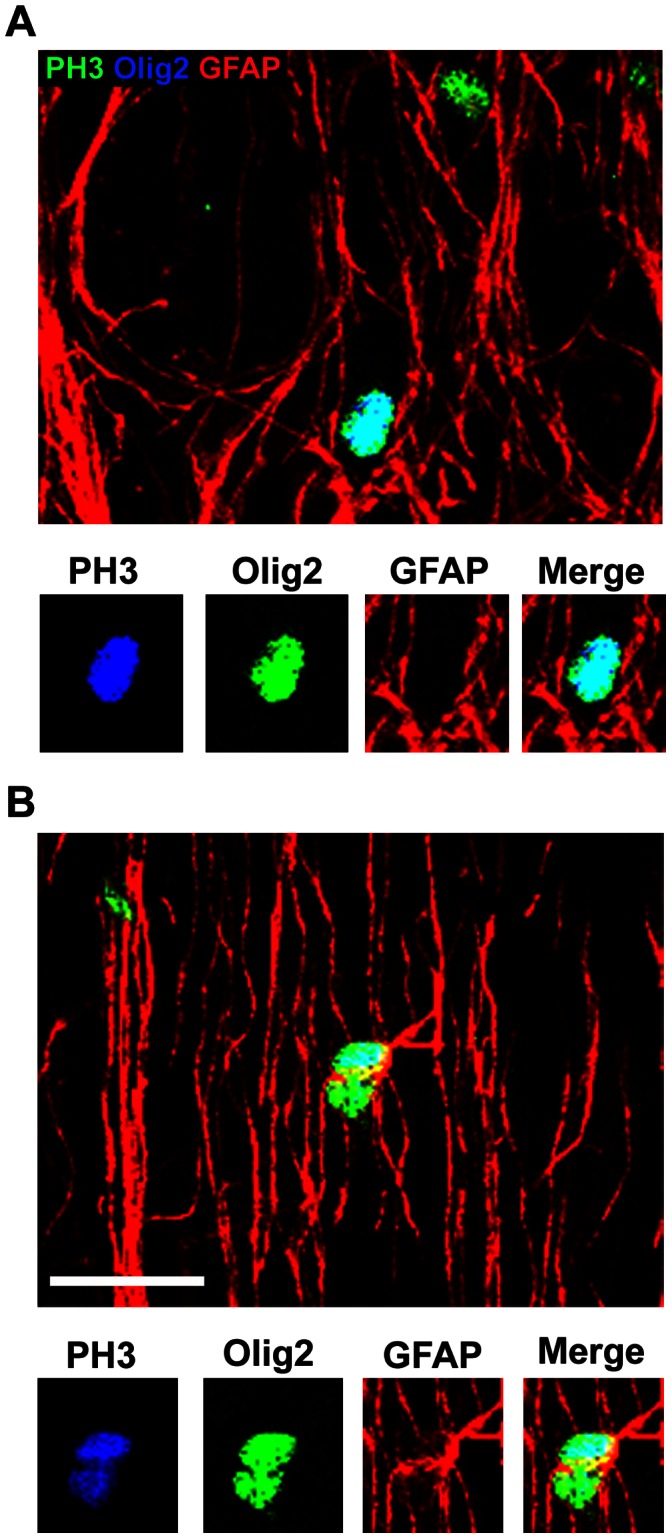
The majority of GFAP-negative mitoses in the prenatal macaque neocortex also express Olig2. *(* ***A*** *)* An example of a mitotic PH3+ precursor cell (blue) that expresses Olig2+ (green) but does not express GFAP (red). Inset panels below show immunoreactivity for each individual antibody. *(* ***B*** *)* An example of PH3+/Olig2+ mitotic precursor cell that expresses GFAP. Inset panels below show immunoreactivity for each individual antibody. Scale bar = 20 µm.

**Figure 15:**
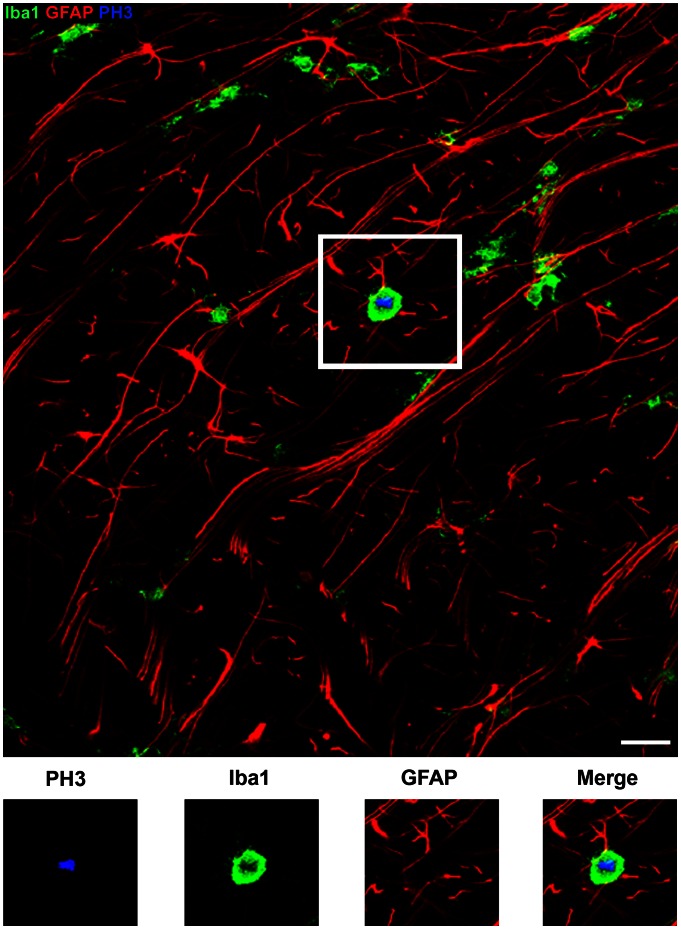
Iba1+ mitotic microglia do not express GFAP. Images show Z-stack projection images taken in the E80 macaque occipital cortex immunostained for PH3 (blue), Iba1 (green) and GFAP (red). Inset panels below show immunoreactivity for each individual antibody. The PH3+ mitotic cell expresses the microglial marker Iba1, but does not express GFAP. GFAP+ processes from nearby cells can be seen coursing near the microglial cell. Approximately 10% of the GFAP-negative mitotic cells in the prenatal macaque neocortex expressed Iba1. Scale bar = 20 µm.

Our data showed that all Pax6+ cells and all Tbr2+ cells in the dorsal neocortex expressed GFAP. These results suggest that all neural precursor cells in the dorsal neocortex that produce excitatory projection neurons express GFAP, especially in light of recent findings indicating that projection neurons destined for each cortical layer arise from Tbr2-expressing precursor cells [64], [65]. Our next set of experiments was designed to phenotype the GFAP-negative mitoses in the dorsal neocortex. We tested whether the GFAP-negative mitoses in the dorsal neocortex expressed the neural transcription factor Mash1/ASCL1, the oligodendrocyte lineage marker Olig2, or the microglial marker Iba1. ASCL1 labels precursor cells in the dorsal human neocortex [3], and ASCL1+ precursor cells may produce cortical interneurons in the dorsal neocortex [3], [39], [66], [67]. We found that ASCL1+ mitoses were numerous in the dorsal neocortex of the macaque, and that the majority of ASCL1+ mitoses were GFAP+ (48/51 cells, Fig. 13). However, there were also a number of ASCL1+ mitotic cells that did not express GFAP, and ASCL1+ mitoses constituted 68% of the GFAP-negative mitoses in the dorsal neocortex (n = 28/41 mitoses). A similar proportion of the GFAP-negative mitoses in the SVZ expressed Olig2, a transcription factor expressed by cells in the oligodendrocyte lineage (n = 27/41 mitoses, Fig. 14). Because of the similar proportions of GFAP-negative precursor cells that expressed ASCL1 or Olig2 we examined ASCL1/Olig2 co-expression in the proliferative zones. We found that most ASCL1+, GFAP-negative mitoses also expressed Olig2 (86%, 24/28). These data are consistent with the concept that some precursor cells in the macaque dorsal neocortex have the potential to generate both neurons and/or oligodendrocytes as has been reported for fetal human neocortex [67]. Finally, we found that another subset of the GFAP-negative precursor cells, roughly 10% (2/21), expressed the microglial marker Iba1 (Fig. 15). Together these data show that GFAP-expressing precursor cells in the dorsal neocortex include all Pax6+ and Tbr2+ neural precursor cells in the dorsal neocortex, and demonstrate that the GFAP-negative mitotic cells include ASCL1+ interneuronal precursor cells, oligodendroglial precursor cells, and microglia.

### GFAP is Expressed by a Smaller Proportion of Mitotic Precursor Cells in the GE

**Figure 16:**
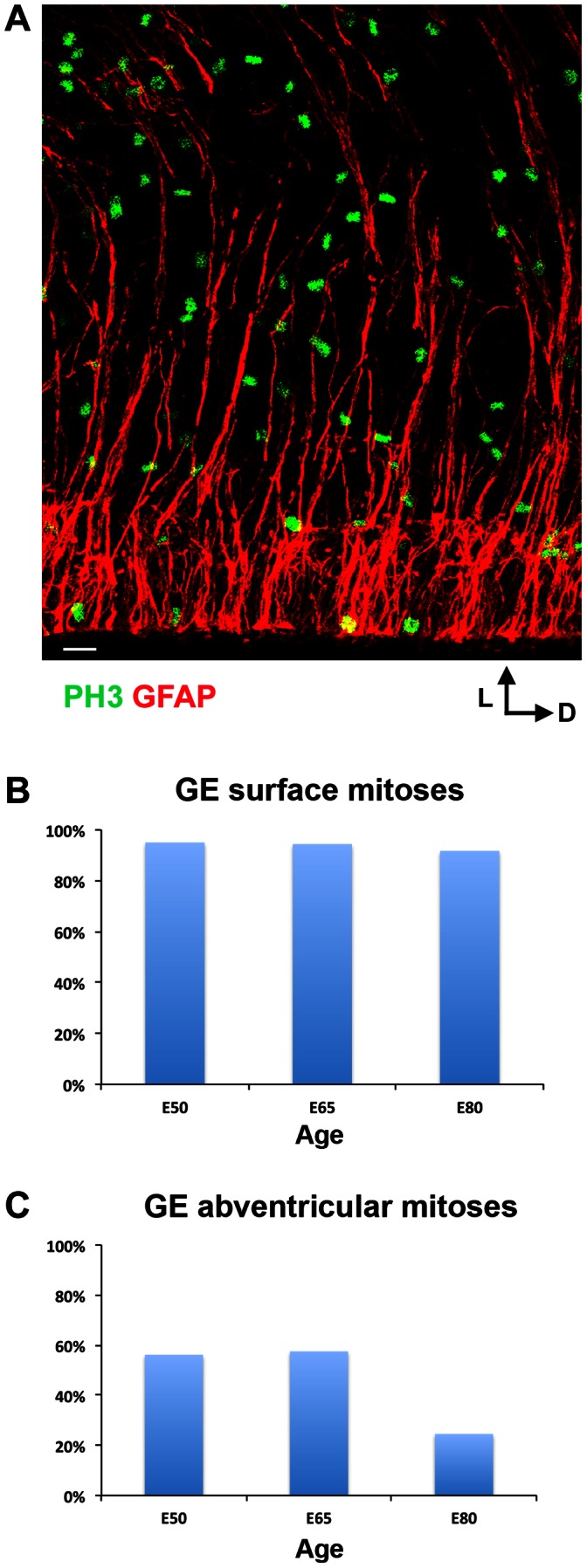
Nearly all mitotic cells at the ventricular surface of the ganglionic eminence (GE) express GFAP, while far fewer abventricular mitoses express GFAP in the GE. A coronal section through the E80 macaque GE immunostained with PH3 (green) and GFAP (red). Over 90% of mitoses at the ventricular surface of the GE were GFAP+ at E50, E65 and E80. In contrast, less than 60% of abventricular mitoses in the GE expressed GFAP at E50 and E65, and the proportion fell to less than 24% at E80. GFAP immunoreactivity was not as dense in the subventricular zone of the GE compared to that in the neocortical SVZ. Scale bar = 20 µm.

We next quantified the proportion of mitotic precursor cells undergoing division in the GE that expressed GFAP at the surface of the ventricle, and away from the ventricle within the SVZ. We immunostained sections of E50, E65, and E80 macaque forebrain with antibodies against GFAP, PH3, and ASCL1. We noted strikingly different patterns of GFAP-IR in the macaque GE compared to that we observed in the dorsal neocortex. Whereas GFAP-IR in the dorsal neocortex produced a dense pattern of labeled cells and fibers extending from the ventricle to the pia, we noted a sharp drop in the density of GFAP-IR from the VZ to the SVZ in the macaque GE (Fig. 16A). There was also a lower proportion of mitotic precursor cells that expressed GFAP in the GE, especially in the SVZ. In the dorsal neocortex, all surface dividing precursor cells expressed GFAP throughout neurogenic stages (n = 413 mitoses). In contrast, GFAP was expressed by most, but not all, surface dividing precursor cells in the GE. GFAP was expressed by 95% of GE surface mitoses at E50 (n = 38/40 mitoses), 94% of GE surface mitoses at E65 (n = 50/53), and 92% of GE surface mitoses at E80 (n = 44/48, Fig. 16B). The proportion of abventricular mitoses in the GE that expressed GFAP was lower in comparison to that in the dorsal neocortex. In the dorsal neocortex 70% or greater of the abventricular dividing precursor cells in the iSVZ and oSVZ expressed GFAP between E50 and E80 (n = 531 mitoses). In contrast, fewer than 60% of abventricular mitoses expressed GFAP in the GE between E50 and E80. GFAP was expressed by 56% of abventricular mitoses at E50 (n = 23/41 mitoses), 58% of abventricular mitoses at E65 (n = 46/80), and only 24% of abventricular mitoses at E80 (n = 29/122, Fig. 16C).

We next examined the proportion of GE mitoses that expressed ASCL1. We observed numerous abventricular mitoses in the GE that expressed ASCL1, as we noted in the dorsal neocortex. However, the proportion of ASCL1 mitoses that co-expressed GFAP was markedly lower in the GE. Only 30% of the ASCL1+ mitoses in the GE expressed GFAP (n = 15/50 mitoses), compared to 94% in the dorsal neocortex (Fig. 13C). Together these results demonstrate that the GFAP+ astroglial cell lineage encompasses a much larger proportion of precursor cells in the dorsal neocortex than it does in the GE.

## Discussion

How the diverse array of cell types that populate the cerebral cortex is generated remains a central question in studies of cortical development. The concept that different cortical cell types derive from distinct precursor cells was proposed over 100 years ago, and multiple lines of evidence have shown conclusively that neocortical precursor cells express different markers and exhibit different morphology and fate potentials. For example, Gotz and colleagues identified subsets of precursor cells in the prenatal proliferative zones [68], [69]. There are also temporal and spatial changes in the expression of genes/transcription factors by precursor cells during neurogenesis that are related to the sequential production of cortical neuron subtypes [31], [70], [71]. Recent work has also revealed sub-lineages of radial glial cells in the dorsal neocortex that produce distinct subtypes of excitatory projection neurons [10].

We focused our study on precursor cells that were actively dividing during specific stages of neocortical development. This approach centers attention specifically on cortical precursor cells, and avoids the potential for lumping together distinct cell types that may temporarily share expression of markers or transcription factors. For example, in the developing rodent neocortex Tbr2 expression is maintained in newborn neurons for a short period as Tbr1 expression is upregulated in post-mitotic cortical neurons [30]. Selecting actively dividing precursor cells for analysis guarded against the possibility that the same pattern of co-expression occurs in the primate cortex, eliminating newly born neurons from our analysis.

### Neocortical Precursor Cells that Produce Projection Neurons are Characterized by GFAP Expression

We identified all mitotic precursor cells in the dorsal neocortex of the prenatal macaque with 4A4 and/or PH3 antibodies. We had previously determined that 4A4 labels all dividing cells in the prenatal macaque cerebral cortex [34]. We asked what proportion of the dividing cells expressed GFAP, and what proportion co-expressed Pax6, Tbr2, Mash1/ASCL1, Olig2, or Iba1. In this way we could distinguish precursor cells for excitatory neurons (Pax6+, Tbr2+), interneurons (ASCL1+), oligodendrocytes (Olig2+), and microglia (Iba1+). We show here that all mitotic cells in the dorsal telencephalon of the prenatal macaque that express the transcription factors Pax6 and/or Tbr2, also express the astroglial marker GFAP. These data demonstrate that the neural precursor cells that produce excitatory cortical neurons share in common the expression of GFAP and thus exist within the same broad lineage of GFAP+ precursor cells. This is consistent with our previous finding that all mitotic cells at the surface of the ventricle in the prenatal rat neocortex express the radial glial cell marker 4A4 [28]. Furthermore, our data predicts that the Cux2+ and Cux2-negative precursor cells that produce excitatory neurons for the upper and lower layers of the mouse neocortex [10] would also exist within the same broad class of precursor cells. Thus our data suggest that the heterogeneity of precursor cell types in the proliferative zones, especially in the VZ, develops within the astroglial cell class.

We find that all mitotic cells at the surface of the ventricle in the macaque dorsal telencephalon express GFAP. At first glance, our data may seem to contradict previous work of Levitt and Rakic who reported the existence of GFAP positive and negative precursor cells in the prenatal macaque neocortex [13], [14]. Our data confirm their findings, but we show that all GFAP-negative precursor cells undergo division away from the ventricle in the SVZ. In addition, we believe that technical considerations may explain some of these differences. Levitt and Rakic used thorough analysis of GFAP immunostained tissue with light microscopy and EM [13], [14], whereas we analyzed GFAP expression with confocal microscopy. We used cell-specific markers, which were not yet available for the earlier studies, to identify actively dividing mitotic cells (anti-PH3), the soma of mitotic cells (anti-4A4), and neocortical precursor cell types (anti-Pax6, Tbr2, ASCL1, Olig2, Iba1) to differentiate between distinct precursor cell types. These cell-type specific markers imaged with confocal or multiphoton microscopes facilitate relatively rapid, straightforward cell identification and co-localization studies.

We phenotyped the GFAP-negative precursor cells in the SVZ and determined that they include Mash1/ASCL1+ neural precursor cells that Rakic and colleagues previously identified in the dorsal human neocortex [3], Olig2+ oligodendrocyte precursor cells, and Iba1+ mitotic microglia. ASCL1-expressing precursor cells are thought to produce cortical interneurons [3], [66], and appear to do so in both the dorsal neocortex [3], [39], and the GE [72]. We found that GFAP-negative mitoses comprise roughly 13% of mitoses in the iSVZ and oSVZ of the E80 macaque dorsal neocortex (Fig. 8B & 8C). Since our data show that ASCL1+ mitoses represent 68% of the GFAP-negative mitoses in the SVZ, we estimate that the GFAP-negative/ASCL1+ mitoses represent approximately 10% of all precursor cells in the E80 macaque SVZ. Interestingly, Kriegstein and colleagues have found that ASCL1+ and Tbr2+ precursor cells are present in the same lineage in the human dorsal neocortex [39], providing evidence that some of the ASCL1+ cells may have a dorsal origin since Tbr2 expression is restricted to the dorsal neocortex. ASCL1+ precursor cells are present in both the dorsal and ventral telencephalon, but we found that the dorsal and ventral ASCL1+ precursor cells exhibited differential patterns of GFAP expression. While nearly all ASCL1+ dividing precursor cells expressed GFAP in the dorsal neocortex (∼94%), only 30% of the ASCL1+ dividing precursor cells in the GE expressed GFAP. We were not able to determine if the ASCL1+ precursor cells we observed in the dorsal macaque neocortex were generated directly in the dorsal telencephalon or rather were initially produced in the GE and subsequently migrated into the dorsal cortex where they undergo mitosis. But our data suggest that GFAP expression may provide hints. We hypothesize that GFAP+/ASCL1+ precursor cells were produced locally in the dorsal neocortex as indicated by Kriegstein and colleagues [39], while the GFAP-negative/ASCL1+ precursor cells were more likely derived from the GE.

Recent work has reported the existence of a non-radial glial precursor cell in the VZ termed a ‘short neural precursor” [73], [74]. Further work is needed to characterize and distinguish this type of cell using cell specific markers such as Pax6, Tbr2, ASCL1, and Iba1. IP cells and ASCL1+ precursor cells are mitotic, lack pial processes, and are found in the VZ [23], [34], [39], [65], and could therefore be characterized as ‘short’ neurogenic precursor cells. Alternatively, Pax6+ RG daughter cells produced through symmetric RG divisions could initially lack a pial process before growth of the pial process and therefore also fit the ‘short neural precursor’ morphology. In addition, we have found that Iba1+ microglia colonize the VZ, are proliferative, and that some acquire an RG-like morphology, but have a short process oriented toward the pia rather than a long pial process [44]. Nonetheless, we find that all mitotic precursor cells in the dorsal telencephalon express GFAP, with the exception of some ASCL1+ precursor cells, oligodendrocyte precursor cells, and microglia. Thus, our data suggests that ‘short neural precursors’ would most likely be in the GFAP-expressing lineage if they derived from the dorsal cerebrum.

Many of the GFAP-negative precursor cells we identified in the dorsal neocortex originated from subcortical or extracortical sites. For example, many ASCL1+ cortical interneurons may be derived from the GE in primates [3], oligodendrocyte precursor cells originating in the ventral forebrain have been shown to migrate into the dorsal neocortex [6], and microglia originate in the yolk sac [75]. Our data suggest that cortical cells derived from extracortical sites are less likely to express GFAP. Consistent with this idea, our data show that the proportion of GFAP+ precursor cells in the dorsal neocortex is much higher than it is in the GE throughout the ages under study, E50– E150.

In conclusion, we demonstrate that all mitotic precursor cells at the ventricle of the dorsal neocortex express GFAP, and that all Pax6+ and all Tbr2+ precursor cells express GFAP. These data demonstrate that neural precursor cells for excitatory projection neurons in the dorsal neocortex exist within the astroglial lineage. This indicates that the astroglial lineage produces neurons throughout life [24], and suggests that the heterogeneity of neural precursor cells in the dorsal neocortex develops within the astroglial lineage.

## Methods

### Animals

All experiments were conducted in compliance with the NIH Guide for Care and Use of Laboratory Animals and were approved by the Institutional Animal Care and Use Committee of the University of California at Davis (Protocol #16368). Macaque tissue (*Macaca mulatta*) was collected by the laboratory of Dr. David Amaral for another study, and brain tissue was provided for this study as a generous gift. All procedures performed on macaques were approved by the Institutional Animal Care and Use Committee of the University of California, Davis (protocol # 12139), and strictly adhered to National Institutes of Health policies on primate animal subjects. Prenatal macaque brains used in this study were aged embryonic day (E) 50, E65, E80, E100, and E150. Pregnant macaques lived in 2000 m^2^ enclosures and were maintained on a diet of fresh fruit, vegetables and monkey chow (Lab Diet #5047, PMI Nutrition International Inc., Brentwood, MO), with water available ad libitum. All attempts were made in terms of social housing and enriched diet to promote the psychological well-being of the animals in accordance with recommendations made by the Weatherall report, “The use of non-human primates in research.” On E50, E65, E80, E100 or E150, caesarian surgeries were performed by surgical veterinarians at the California National Primate Research Center (CNPRC). Fetuses were removed, anesthetized and transcardially perfused with saline followed by 4% paraformaldehyde in phosphate buffered saline (PFA). Donor mothers were sutured and allowed to recover from the surgery per standard operating procedures at the CNPRC. Fetal human tissue was donated by Dr. Gimenez-Amaya. Parents gave written consent for the fetal tissue to be collected and used for research. Tissue samples were anonymized for research purposes and did not have any identifiable subject information attached. The biological samples used in this study were provided after the approval by the Ethical Committee of the hospital at the Universidad Autónoma de Madrid.

### General Tissue Preparation

After transcardial perfusion with PFA, brains were removed, post-fixed for at least 2 hours in PFA, and cryoprotected overnight in 30% sucrose. Cryoprotected brains were stored at −80**°**C until they were sectioned at 30 µm on a cryostat (Microm) and mounted on glass slides.

### Immunohistochemistry

Antigen retrieval was performed by boiling sections for 10 minutes in 10 mM Citrate Buffer pH 6.0 containing 10 mM citric acid (Fisher) and 0.5% Tween-20 (Acros) in a microwave. Sections were incubated in blocking buffer that contained 10% Donkey Serum (Millipore), 0.1%Triton X-100 (Acros), and 0.2% gelatin (Acros) for 1 hour at RT. Sections were then incubated overnight at RT in primary antibody buffer containing 2% Donkey Serum, 0.02% Triton X-100, and 0.04% gelatin and primary antibodies that included mouse anti-4a4 1:500 (MBL Intl), mouse anti-GFAP 1∶400 (Sigma), mouse anti-Pax6 supernatant 1∶1 (DSHB), mouse anti-Mash1/ASCL1 1:50 (BD Biosciences), rabbit anti-Tbr2 1:500 (Abcam), rabbit anti-Pax6 1:100 (Covance), rabbit anti-GFAP-delta 1∶500 (Abcam), goat anti-GFAP 1∶50 (Santa Cruz), goat anti-Olig2 1:500 (R&D), goat anti-Iba1 1:100 (Abcam), and/or rat anti-GFAP 1∶50 (Virginia Lee, University of Pennsylvania). Sections were rinsed, and incubated for 1–2 hr at RT in secondary incubation buffer containing DAPI 1∶500 (Roche), 2% Donkey Serum, 0.02% Triton X-100, 0.04% gelatin and fluorescently conjugated secondary antibodies (Jackson Labs) including donkey anti-mouse (1∶200), donkey anti-rabbit (1∶200), donkey anti chicken (1∶200), donkey anti-goat (1∶200), and/or donkey anti rat (1∶200). Sections were rinsed in PBS and then coverslipped with Mowiol.

### Confocal Microscopy

Sections of brain tissue were viewed and imaged on a Fluoview FV1000 Confocal Microscope (Olympus). Analysis and quantification were performed on the Olympus software and in Photoshop CS3 (Adobe). Images were cropped, contrast adjusted and false color applied in Photoshop.
